# Seabird Prey in Space and Time: Combining Multiple Datasets to Model the Distribution of Sandeel (*Ammodytes marinus*) and Sprat (*Sprattus sprattus*) in the North Sea

**DOI:** 10.1002/ece3.74570

**Published:** 2026-10-29

**Authors:** Sylvan Benaksas, Christopher Lynam, Francis Daunt, Chibuzor Christopher Nnanatu, Jeroen van der Kooij, Thomas J. Webb

**Affiliations:** ^1^ Ecology & Evolutionary Biology, School of Biosciences, University of Sheffield Sheffield UK; ^2^ Centre for Environment, Fisheries and Aquaculture Science, Lowestoft Laboratory Suffolk UK; ^3^ UK Centre for Ecology & Hydrology, Bush Estate Midlothian UK

**Keywords:** Bayesian modelling, INLA, sandeel, spatial modelling, species distribution modelling, sprat

## Abstract

Many seabird populations along the UK's East Coast have declined over the past three decades. A key concern is reduced availability of their primary breeding season prey, sandeel ( *Ammodytes marinus* ). While some seabirds have partially switched to sprat ( *Sprattus sprattus* ), this is not universal. Studying these predator–prey dynamics requires fine‐scale estimates of prey distributions that are both spatially and temporally resolved—yet such data are limited, especially for sandeel due to sampling challenges. We evaluate the utility of large‐scale ICES bottom trawl surveys to model sandeel distribution, combining them with winter dredge surveys designed specifically for this species. For sprat, we combine bottom trawl data with the Herring Acoustic Survey. Using hierarchical Bayesian spatiotemporal models (R‐INLA), we estimate the biomass of small and medium sandeel (≤ 16 cm) and sprat across the entire North Sea from 2000 to 2020, accounting for spatial and temporal dependencies. We validate our sandeel models by comparison with other modelling approaches and ICES stock assessments. Results reveal marked regional variation: sandeel increased in Area 3 (Norwegian EEZ), declined in Areas 1 and 2 and fluctuated in Area 4. Sprat biomass increased over time, with a modest northward expansion. This study provides the first North Sea‐wide, fine‐scale, multi‐decadal distribution models for these two key seabird prey species and identifies important regional drivers of their populations.

## Graphical abstract

Forage fish are key prey and commercial species in the North Sea ecosystem, but estimates of their biomass over detailed spatial and temporal scales is currently limited. To fill this vital research gap we employ Hierarchical Bayesian spatiotemporal models of sandeel ( *Ammodytes marinus* ) and sprat ( *Sprattus sprattus* ) in the North Sea, providing fine‐scale predictions of biomass over two decades and identify key environmental drivers.

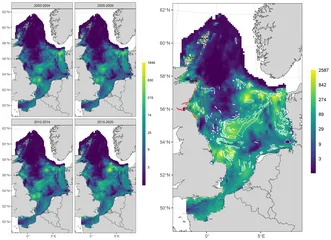

## 1 Introduction

Seabirds are among the most endangered bird groups globally and rank among the most threatened vertebrates (Phillips et al. 2023). They face multiple anthropogenic pressures, including invasive species, bycatch, climate change and overfishing (Dias et al. 2019). In the North Sea, many seabirds feed on small, schooling pelagic fish species—commonly known as forage fish—that primarily consume zooplankton (Alder et al. 2008). Overfishing of these prey places commercial fisheries in direct competition with seabirds, exacerbating concerns about prey availability amid declining seabird populations (Grémillet et al. 2018). Because forage fish exhibit rapid “boom and bust” population dynamics due to their short lifespans and low trophic position (Cury et al. 2000; Engelhard et al. 2014), understanding their spatiotemporal fluctuations is critical for assessing their availability to predators.

In the North Sea, many seabird populations have declined in recent decades, raising conservation concerns (Burnell et al. 2023). These declines are likely driven by a combination of climate change (Burton et al. 2023), commercial fishing and prey availability (Cook et al. 2014; Searle et al. 2023). Such impacts are particularly acute during the breeding season, when seabirds are restricted to foraging near colonies and energy demands are high (Rindorf et al. 2000). Local prey shortages have been associated with widespread breeding failures in the North‐East Atlantic, with birds travelling further and expending more energy to forage (Fayet et al. 2021). Robust understanding of these predator–prey relationships requires high‐resolution data on the distribution of both seabirds and their key prey across sufficient spatial and temporal scales.

While seabird foraging distributions in the western North Sea are well studied (Waggitt et al. 2020; Wakefield et al. 2017), fine‐scale distribution data for their forage fish prey remain limited (Searle et al. 2022; Wright et al. 2019). Past studies often used environmental proxies or coarse regional fish stock assessments (Cook et al. 2014; Lynam et al. 2017), limiting ecological inference. In the Western North Sea, seabird diets during the breeding season are dominated by two key forage fish: lesser sandeel ( *Ammodytes marinus* ), hereafter sandeel and European sprat ( *Sprattus sprattus* ) (Anderson et al. 2014; Wanless et al. 2018). Both have experienced significant pressure from overfishing (Dickey‐Collas et al. 2010; Sherman et al. 1981), with sandeel becoming the focus of the region's largest fishery after sprat collapsed in the 1980s (Alder et al. 2008; Frederiksen et al. 2004).

Sandeel populations have declined markedly since the early 2000s and have not recovered (ICES 2022a). In addition to fishing pressure, they are also vulnerable to climate change (Carroll et al. 2017; Lynam et al. 2013) and increased predation from piscivorous fish (Engelhard et al. 2014). Their dominance in seabird diets has decreased, particularly in northern colonies, where sprat have partially replaced them, however, despite these declines, sandeel remain critical prey for many predators (Wilson and Hammond 2019; Ransijn et al. 2021; Searle et al. 2023). In recognition of their ecological importance, a fishery closure was implemented off eastern Scotland in 2000 (Rindorf et al. 2000). Results have been mixed: only black‐legged kittiwakes ( *Rissa tridactyla* ) showed improved breeding success (Greenstreet et al. 2006; Searle et al. 2023). Studying such predator–prey dynamics requires finer‐scale information than is provided by current sandeel stock assessments, which are aggregated into seven broad management areas (ICES 2022a). Dredge and grab surveys do provide valuable data, but this is highly localised (Greenstreet et al. 2010), whereas acoustic surveys—which are regional in extent—are generally not utilised for sandeel in the North Sea outside of Norway, as they lack swim bladders and are difficult to detect acoustically (Johnsen et al. 2009). Consequently, spatial information has relied on fisheries‐dependent data (Jensen et al. 2011), and large spatial gaps remain in our understanding of their distribution.

Sprat are at the northern edge of their geographic range in the North Sea and remain below historical biomass; however, with high uncertainty for estimates pre‐1980, despite reduced fishing pressure (ICES 2020). They now appear more frequently in predator diets in the southern and western North Sea (Ransijn et al. 2021; Wanless et al. 2018), but a lack of fine‐scale spatiotemporally detailed data hampers interpretation. Although managed as a single stock, their summer distribution has only been modelled at fine scales in southern areas (Ransijn et al. 2021), excluding key foraging areas for seabirds such as Shetland and the Isle of May. Sprat are better sampled than sandeel, being reliably caught in acoustic and bottom trawl surveys, yet a unified, spatiotemporal analysis of these data is lacking.

Recent efforts to predict sandeel distributions have used local surveys with environmental covariates (Langton et al. 2021), but limited spatial coverage and no temporal component introduces high uncertainty when extrapolating to unsampled regions. Similarly, sprat habitat models have either been coarse, seasonally limited or lacked biomass estimates (Lenoir et al. 2011; Lindegren et al. 2022; Schickele et al. 2021).

Lindegren et al. (2022) identified the absence of fine‐scale spatiotemporal biomass models of forage fish as a key knowledge gap. Filling this gap is a necessary prerequisite to allow detailed, local studies which link prey availability to seabird demographic parameters (Smout et al. 2013) to be scaled up to fully understand the causes and ecological consequences of seabird declines at regional scales. Here, we address this by combining multiple fishery‐independent survey datasets using a Bayesian spatiotemporal modelling framework to provide fine scale, temporally resolved models of sandeel and sprat biomass for the entire North Sea ecoregion. Our approach combines data from targeted but spatially restricted winter dredge survey for sandeel, and forage fish targeted but temporally restricted Herring Acoustic Survey (HERAS) for sprat, with broad‐coverage bottom trawl surveys such as the International Bottom Trawl Survey (IBTS) and Beam Trawl Survey (BTS). While trawl surveys have traditionally been viewed as unreliable for sandeel due to low catchability (Wright et al. 2019), their broad spatiotemporal extent provides valuable insight into sandeel distribution patterns. By pooling datasets, we aim to produce the first spatiotemporal models of sandeel and sprat biomass across the North Sea at a scale which can be employed in future models of predator distribution and dynamics.

## 2 Materials and Methods

### 2.1 Fish Survey Data

We combined datasets from multiple fisheries‐independent surveys to model prey fish biomass in the Greater North Sea (Figure 1), focusing on the years 2000–2020. Trawl and dredge data were downloaded from the ICES DATRAS portal (https://datras.ices.dk/WebServices/Webservices.aspx). This period excludes early data (1990–1999) where annual number of trawls containing sandeel numbered 5 or below, raising concerns about inconsistent recording practices. As we were interested in the prey available to seabirds during the breeding season, an upper sandeel length limit of 16 cm was selected for small to medium sized bird species based on a review of available diet literature of seabird chick provisioning (Harris and Hislop 1978; Harris and Wanless 1986; Thaxter et al. 2013; Wanless et al. 2004, 2005, 2018). Different seabirds across different time periods target young‐of‐the‐year fish or 1+ group sandeel, with a general transition to young fish around June as the older fish become less available, presumably as they begin to bury in the sediment for winter (Daunt et al. 2008). However, given the variability in size‐at‐age of sandeel over recent decades (MacDonald et al. 2019), we did not age‐stratify our models and included all biomass within the edible size range. We repeated our analyses including all sandeel sizes (up to *c*.25 cm) which resulted in broadly similar results, indicating no major bias from this size cutoff. No size filtering was required for sprat as they rarely grow larger than 16 cm.

**FIGURE 1:**
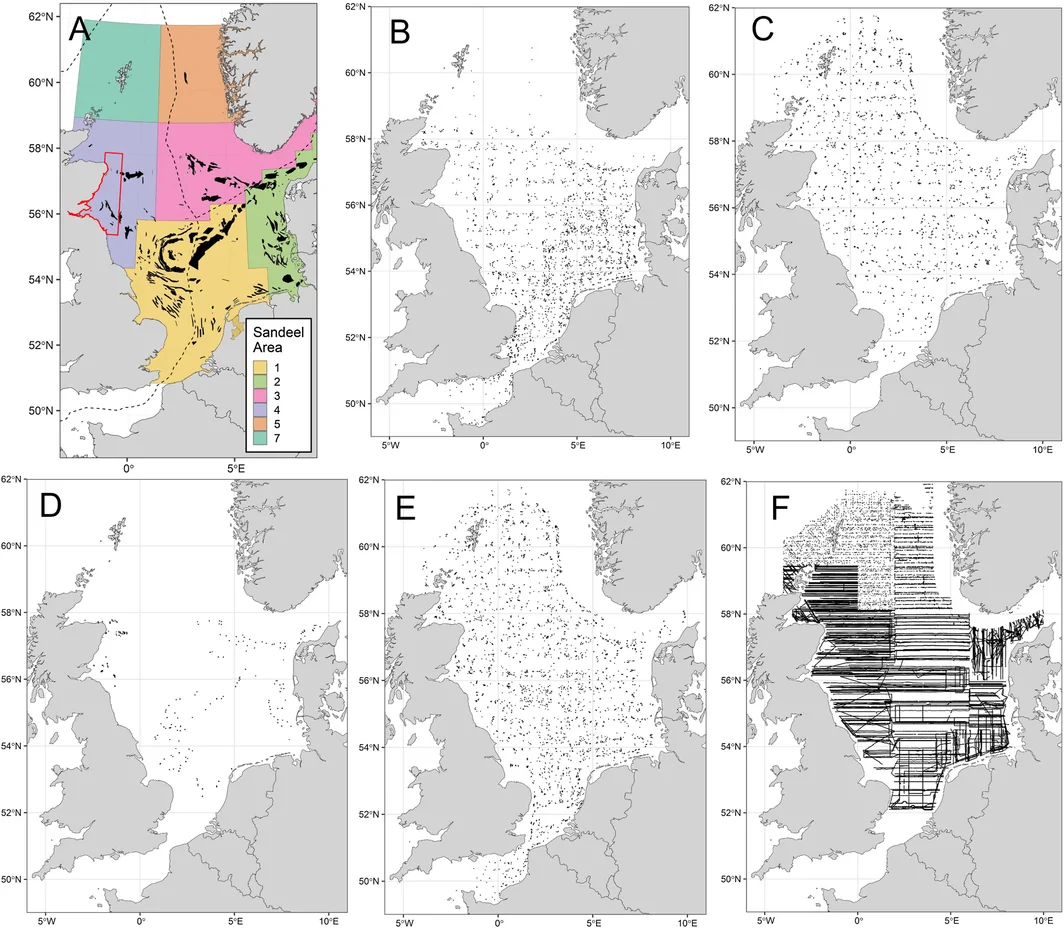
(A) Map of sandeel management areas by colour; the solid black shapes denote the core sandeel fishing grounds estimated from Jensen et al. (2011), the red line shows the area closed to sandeel fishing and the dashed lines show the EEZ of the UK, Norway and the EU. Map of survey locations in the North Sea for (B) BTS, (C) Q3 IBTS, (D) Sandeel dredge survey, (E) Q1 IBTS data and (F) HERAS. The HERAS survey map shows the dataset after thinning the northern absence points.

The International Bottom Trawl Survey (IBTS) and Beam Trawl Survey (BTS) use otter and beam trawls, respectively, to target demersal fish. These gear types are not optimised for small pelagic species like sandeel, whose catchability is low due to small size, thin shape and variable burial in sediment (Fraser et al. 2007). Consequently, this rich data source has until now only been used for sprat and not been applied in the study of sandeel. Accounting for catchability issues, we applied length‐specific gear efficiency corrections from Walker et al. (2017) to adjust biomass estimates from IBTS and BTS accordingly, for both sandeel and sprat. We cannot account for uncertainties in the distribution of fish in the water column or sediment for sandeel. However, acoustic work in Norway has shown that sandeel schools form continuous distributions from the seabed up into the water column, suggesting that they are likely to be at least partially sampled by demersal trawls (Johnsen et al. 2017). Post‐juvenile sprat tend to be closer to the seabed during the day (Plirú et al. 2012), and this is evident from the much greater catch rates of sprat in the IBTS than for sandeel.

Given that sandeel biomass is often underrepresented in standard Q3 trawl surveys (July–September), we incorporated additional winter data: Q1 IBTS and the dedicated sandeel dredge survey (Figure 1D,E). The dredge survey is specifically designed for targeting sandeel; however, this survey does have a limited spatial and temporal coverage across the entire North Sea. Although surveys conducted in a different season potentially introduce uncertainty into our models by decoupling from summer environmental conditions, this is minimised by the fact that sandeel are inactive and buried in the sediment from late summer and throughout the winter surveys (Winslade 1974), and more generally sandeel post‐settlement have low mobility and do not perform migrations (Jensen et al. 2011). Sandeel surveyed in winter are thus likely to be in the same location that they were found the previous August, and so it is a reasonable assumption that they experienced those August environmental conditions just before they entered the sediment to overwinter. We therefore assigned the location and biomass values from the winter surveys to the previous summer, with associated August environmental conditions. Overall, the composite dataset benefits from the combination of high‐quality dredge survey data with the extended spatial and temporal coverage of the bottom trawl surveys.

Dredge surveys, using a modified scallop dredge, began in 2008 (area 4) and 2012 (areas 1r–3r) and methods are described in (Johnsen and Harbitz 2013). Based on published estimates (Johnsen and Harbitz 2013; Langton et al. 2021; Mackinson et al. 2005), we assumed 8% dredge efficiency and applied a biomass raising factor of 12.5. No length‐based catch efficiency has been found in the sandeel dredge survey with the exception that 2+ sandeel are less reliably caught (Johnsen and Harbitz 2013). This is likely not an issue for our analysis because our data cut at 16 cm and smaller is unlikely to include 2+ fish in significant proportions. Efficiency was thus assumed constant across length classes. There is likely some sandeel mortality that occurs between Q3 and the winter surveys which we have not adjusted for, and the winter surveys are therefore a conservative estimate of biomass. Sandeel biomass was estimated from fish length using length‐weight relationships that account for seasonal body condition (Rindorf et al. 2016).

Sprat biomass was augmented using the HERAS survey (Figure 1), which provides water column coverage in late June and July across the entire North Sea (2009–2020). Full details of HERAS are provided by ICES (2022b), but in common with other acoustic surveys it is carried out using echosounders mounted on the hull, a drop‐keel or on a towed body. Because of the mounting position and noise environment at the surface, the top *c*.8–10 m of the water column is unobservable, but while this is a potential source of bias, this is minimised by the fact that the diel vertical migration of sprat means their distribution during daytime is typically deeper than this (Bernreuther et al. 2013). The ping rate of the echosounder is on average once every second and the rebounded backscatter values, Nautical Area Scattering Coefficient (NASC), are averaged over a square nautical mile along the acoustic transects. Fish of different species and lengths have different reflectance properties, and by using species and length specific Target Strength equations (Simmonds and MacLennan 2007), NASC can be converted to abundance and biomass. Pelagic trawls are typically conducted to derive details on the species composition as well as biological information including length distributions.

The HERAS survey grid in the North Sea is divided into strata for data processing, which were updated in 2017 (ICES 2022b) and we use these for all years, 2009–2020. HERAS acoustic and trawl data were downloaded from the ICES acoustic database (https://acoustic.ices.dk/submissions). Scotland, the Netherlands and Norway provided backscatter (NASC) values at species level for sprat and herring ( *Clupea harengus* ). However, other nations did not, in which case associated trawl data were used to partition the acoustic data by species. For several older surveys, accompanying pelagic trawl data were missing: specifically for Norway (2010–2014 and 2017), Denmark (2010–2015) and Germany (2010–2016). To estimate species composition in these cases we used IBTS trawls from July and August. This is imperfect because IBTS trawls typically only sample up to ~5 m above the seabed rather than the water column and there is a several week time‐lag between the surveys. To investigate whether we could use IBTS data to supplement missing pelagic trawl data, we compared the percentage composition by weight of clupeids per trawl for IBTS and HERAS annually across each strata for years and areas for which we have HERAS pelagic trawls. We found the species composition between these was moderately correlated (Pearson's correlation, *r* = 0.59). Accordingly, we used the IBTS trawl data to supplement the HERAS survey when we lack pelagic trawls.

Germany uploaded data labelled as clupeids, but did not distinguish NASC values between sprat and herring. Denmark did not differentiate NASC values between clupeids, mackerel ( *Scomber scombrus* ) and gadoids. Because mackerel and sandeel lack a swim bladder, they are poorly detected at the target echosounder frequency of 38 Hz (the standard used in HERAS), so both species were ignored when partitioning the “clupeid” and other non‐specific backscatter by the trawl catches (van der Kooij et al. 2016). Acoustic surveys are mostly done during the daylight when clupeids aggregate in dense schools. During these hours, most gadoids are found in the demersal or “acoustic dead” zone (Doray et al. 2021; Mackinson et al. 2005), and so are usually also disregarded during NASC allocation, the exception being whiting which may be active in the water column. Non‐whiting gadoids were therefore excluded in acoustic partitioning. The Danish survey is conducted continuously over 24 h and data recorded during the night were removed to avoid the inclusion of gadoid backscatter. Large parts of the northern North Sea around the Shetland Islands and east to the coast of Norway detected no sprat as this is likely beyond sprat's northern range, and so to avoid biasing the model we thinned the data points and only included a random 10% of these very northern absence records. Sprat biomass was estimated using derived seasonal length‐weight relationships from Wilhelms (2013). The total biomass (kg) per species was then summed per trawl.

As there was no relationship between sandeel biomass and swept area for any of the surveys (Pearson correlation) we did not include swept area in the model. As each survey had a standard trawl distance, the inclusion of a fixed effect for survey type in the model captures survey‐specific difference in swept area as well. However for sprat, because the data were joined with the HERAS acoustic survey (see below) which averages biomass estimates over a square nautical mile, IBTS biomass values were raised to biomass per square mile. This was done by dividing the biomass by the swept area in km^2^ and scaling this up to the equivalent value per square nautical mile to make the datasets comparable, making the response variable for sprat equivalent to catch‐per‐unit‐effort (CPUE).

While both acoustic and bottom trawl data are considered reliable for sprat, rather than exclude valuable datasets, we therefore pooled the datasets explicitly within a Bayesian hierarchical model structure to jointly analyse these data. The pooling of acoustic and bottom trawl data has been shown to have variable results (Fiorentino et al. 2013; Kotwicki et al. 2018), with particular challenges reported for the North Sea (Bouleau et al. 2004). These challenges have been attributed to differences in spatial and vertical coverage, catchability, survey design and species, and may require advanced spatial modelling approaches to combine the data adequately (Bouleau et al. 2004; Kotwicki et al. 2018; von Szalay et al. 2007). While this poses a risk of increased uncertainty, we think the potential benefit of increased spatial and temporal coverage justifies testing the utility of combining acoustic and bottom trawl surveys for North Sea sprat. For both species, all biomass estimates were adjusted to 100% gear efficiency, and survey type was included as a fixed effect to account for methodological differences. This approach leverages the complementary strengths of each survey to improve spatial and temporal resolution—particularly for sandeel, for which data coverage is a prime concern—while trying to account for known biases in the data. To our knowledge, this is the first study to combine these data sources into a unified spatiotemporal framework for sandeel. Survey coverage summaries are shown in Table 1.

**TABLE 1:** Summary of survey data sources.

Dataset	Start year	End year	Years	Season	Months	Temporal shift	Data points	Sandeel	Sprat
BTS	2000	2020	21	Summer	July‐Sept	✗	4961	✓	✗
Q3 IBTS	2000	2020	21	Summer	July‐Sept	✗	5334	✓	✓
Q1 IBTS	2000	2019	20	Winter	Jan‐March	✓	6943	✓	✗
Dredge	2008	2020	13	Winter	Nov‐Dec	✓	3979	✓	✗
HERAS	2008	2020	13	Summer	June–July	✗	89, 058	✗	✓

*Note:* For each of the five survey datasets, we provide the range of years that we consider, the season and month in which it is conducted, the number of trawl or detection points in the dataset and whether the dataset was included in the sandeel or sprat model. Temporal shift indicates if a winter sandeel survey was shifted to August.

### 2.2 Environmental Data

The biomass observations were combined with a broad set of environmental covariates to model the spatial and temporal distributions of sandeel and sprat in the North Sea. Given the low dispersal ability of sandeel and their strong association with the sea floor (Holland et al. 2005), seabed habitat characteristics are particularly influential in determining their distribution. We used the seabed sedimentary environment product from Wilson et al. (2018), which maps sediment characteristics across the north‐west European shelf at ~13 km resolution using a combination of survey data and statistical modelling. Specifically, we extracted percentage silt and sand content from this dataset, filling missing data in the Skagerrak region using predictions from Mitchell et al. (2019).

Depth was recorded for most DATRAS trawls, with missing values supplemented from the EMODnet bathymetry layer (https://emodnet.ec.europa.eu/en/bathymetry). Bathymetric slope (in degrees) was derived from the MARSPEC dataset (Sbrocco and Barber 2013) using the R package sdmpredictors (Bosch and Fernandez 2022).

We included mean monthly dynamic oceanographic variables from the Copernicus Marine Service (~7 km resolution: https://www.copernicus.eu/en): mixed layer depth and bottom water velocity from the Physical Reanalysis product, and bottom oxygen, surface net primary production, and phytoplankton concentration from the Biogeochemistry Reanalysis. Bottom velocity was computed as the magnitude of the eastward and northward vector components.

Prior to analysis, we examined all environmental covariates for collinearity using pairwise global Pearson's correlation tests. Depth and bottom temperature showed high correlation (*r* > 0.7). We retained depth over bottom temperature as depth is important for burrowing sandeels (Wright et al. 2000). Covariates were mean‐centred to reduce numerical issues and to ensure interpretability across scales.

### 2.3 Model Fitting

We fitted species distribution models using the IntegratedNested Laplace Approximation (INLA) via the R‐INLA package (Lindgren and Rue 2015; Rue et al. 2009, 2023), a computationally efficient alternative to Markov Chain Monte Carlo (MCMC) methods for Bayesian inference. INLA is especially well suited for spatial and spatiotemporal ecological modelling, yielding realistic fish distribution estimates compared to conventional approaches (Lezama‐Ochoa et al. 2020; Redding et al. 2017).

Survey data for both species was highly zero‐inflated due to habitat specificity and shoaling behaviour. For sandeel, 82.5% of all hauls reported zero catch, rising to 95.5% for the non‐dredge surveys, while 93.5% of points in the sprat dataset recorded zero sprat. Tweedie error distributions can accommodate zero‐inflated, right‐skewed continuous data within a single model framework—unlike hurdle models, which require separate stages and the propagation of uncertainty (Foster and Bravington 2013; Thorson 2018). We applied Tweedie Generalised Additive Models (GAMs) with a log link, incorporating nonlinear responses through smooth functions on selected predictors for sprat and sandeel separately.

We model the response as:

$$y_{i,t}∼\text{Tweedie}\left(\mu_{i,t},\phi ,p\right)$$

Sandeel Model

$$\log\left(\mu_{i,t}\right)=\beta 1X_{i,t}+f\left({\text{Depth}}_{i}\right)+S_{i,t}$$

Sprat Model

$$\log\left(\mu_{i,t}\right)=\beta 1X_{i,t}+f\left({\text{Depth}}_{i}\right)\;+f\left({\text{Phyc}}_{i,t}\right)+f\left({\text{year}}_{t}\right)+S_{i,t}$$

Where *μ*_*i,t*_ is the expected fish biomass at location *i* and time *t*, *ϕ* is the dispersion parameter, *p* is the Tweedie power parameter, *β1* is the vector of regression parameters, *X*_*i*_ is the matrix of covariates at location *i* and time *t*, Parentheses *f*(…) denote smooth terms modelled as random effects and *S*_*i,t*_ represents the yearly spatiotemporal random effect defined via a Gaussian Markov random field using the Matérn SPDE approach at location *i* and time *t*. Backwards model selection was performed using Deviance Information Criterion (DIC) (Spiegelhalter et al. 2014). Model selection was necessary to address multicollinearity and prevent overfitting among our complex suite of environmental predictors. We utilised backward elimination, as starting with the full global model ensures that all covariates and potential interactions are evaluated simultaneously, preserving crucial ecological context that forward selection might prematurely discard. While we acknowledge the inherent limitations of stepwise procedures, this approach allowed us to systematically isolate the most robust, parsimonious drivers of fish distribution. To properly ensure model identifiability the intercept term was excluded and factor variables were fully expanded out into 0/1 dummy variables, with each factor level having a directly estimated coefficient posterior. Survey was included as a fixed effect, encoded as a dummy variable for both models and in the sprat model dummy fixed effects were included for the month variable. For both sandeel and sprat, depth was modelled as a non‐linear effect using a 1D SPDE smoother (similar to the basis‐penalty smoothing approach; Wood 2017), and for sprat, phytoplankton concentration (phyc) was also modelled as a 1D SPDE smoother (Krainski et al. 2018). In the sprat model we applied an autoregressive model of order 1 (AR1) random effect for year. We used penalised complexity (PC) priors for all random effects, which encourage shrinkage and reduces overfitting (Simpson et al. 2017), with prior values detailed in the Supporting Information (see “Prior Specification”). Because the North Sea contains several subpopulations, we did not include a global year effect in the sandeel model, and let the yearly spatiotemporal random effect capture the temporal component of the model.

### 2.4 Model Validation

Models were validated using cross‐validation with an 85%/15% training/testing split on random subsets of the data. Normalised Root Mean Square Error (NRMSE) was calculated to assess out‐of‐sample prediction accuracy of the model, defined as the root mean square error (RMSE) between predicted and observed biomass values, normalised by the range of observed biomass (i.e., maximum minus minimum). This provides a scale‐independent metric that facilitates comparison across species and models (Bell and Schlaepfer 2016). For sandeel, we also compared our results to the outputs of two independent models as well as polygons of sandeel fishing grounds from Jensen et al. (2011) to assess our predictions spatially and temporally. Firstly, we correlated our spatial predictions (hereafter INLA) with those from Langton et al. (2021) (hereafter Langton) across the UK EEZ. As the Langton predictions have no temporal component, we took the sum of predicted biomass per pixel across all 21 years from the INLA predictions, while this removes a lot of variation, it identifies the core sandeel grounds and enables comparison of the two datasets. We then log scaled and standardised each output from 0 to 100 to enable comparison, as the Langton predictions were abundance, measured in numbers. The Langton predictions were at a finer spatial resolution, therefore we rescaled this to a spatial scale of 10 km to match our predictions. We then compared both our results and the Langton predictions, by subtracting the Langton predictions from the INLA predictions and overlaying the sandeel fishing ground polygons to identify the areas where the model predictions diverged and how these related to previously identified core sandeel fishing grounds. Secondly, we correlated our sandeel INLA temporal trend predictions by sandeel management area with those estimated from the ICES annual stock assessment models. For sprat we are not aware of any published summer distributions on the appropriate spatial scale to compare against. We correlated our sprat temporal trend predictions with those estimated from the ICES annual stock assessment model, which is on the scale of the North Sea.

### 2.5 Spatiotemporal Predictions

Annual spatial–temporal predictions were made for the years 2000–2020 using the best model for each species, which included the spatiotemporal random effect. These fine scale predictions were made on a 10 km grid covering the entire North Sea. Predictions were made for July, the earliest summer month for which we had data for both species of fish. For the sandeel model predictions were made using the dredge coefficient, while for the sprat model predictions were made using the IBTS coefficient. As sandeel biomass is habitat‐specific but variable through time, summing predictions over years gives a good indication of the total habitat of the North Sea populations. For consistency we performed the same for the sprat predictions. Temporal trends could also be calculated by summing the predictions across regional populations. For sandeel, this was the sub‐regional sandeel management areas and for sprat this was the entire North Sea. Uncertainty for this regional time series was obtained through posterior sampling using the *inla.posterior.sample* function. This involved sampling 1000 times from the joint posterior distribution for every prediction pixel per year, with each sample realisation summed across regional populations giving 1000 regional time series. This distribution of 1000 regional sums could then be used to calculate 95% credible intervals. As the final sandeel model did not include swept area, the predictions are a relative index of biomass. In contrast the sprat predictions do include swept area and approach absolute estimates. It is therefore not appropriate to compare the biomass estimates, but only possible to compare the general patterns and maps between the species. All predicted fish distributions datasets generated in this study are publicly available (see Data Availability Statement).

### 2.6 Accounting for Spatial and Temporal Autocorrelation

Accounting for spatial and temporal dependence is a central concern for a spatiotemporal model, given that almost all ecological data will contain spatial and temporal autocorrelation, and failing to take account of this can lead to inaccurate model parameters, less accurate predictions and unrealistically small estimates of error (Václavík et al. 2012). However, this can also be a highly informative property and when modelled properly can greatly improve a model's predictions (Dormann 2007). INLA accounts for spatial correlation by using the stochastic partial differential equation (SPDE) method, by approximating a continually indexed zero‐mean Gaussian Markov Random Field (GMRF), where a Matérn covariance function is applied across the surface (Bakka et al. 2018). The SPDE with Matérn correlation is applied across a “mesh” generated using a Delauney triangulation covering the study region (Figures S1, S2). The configuration of the mesh can greatly alter the results of an INLA SPDE model (Verdoy 2021). In general, the mesh needs to be specified based on the underlying data of the model, which depends on the spatial spacing of the response dataset and the spatial resolution of the environmental raster layers (Bakka et al. 2018). Ideally, the step length (maximum triangle edge length) should be as close to the spatial resolution of the underlying environmental datasets so that it can effectively capture the spatial heterogeneity in the environment. However, there is also a trade‐off of computational efficiency as increasing the mesh resolution will increase model running time. For these reasons, we used a mesh step length of 30 km for sandeel and 40 km for sprat, balancing computation time and the need to capture the spatial scale of population drivers. The mesh is clipped to the North Sea marine area using a high‐resolution coastline and the North Sea roundfish areas as approximate cut‐offs across sea; this provided a northern sea limit and avoided including the deep Norwegian Trench. A sea delineated mesh area was used to avoid estimating the spatial effect across land. The spatiotemporal random effect is controlled by four hyperparameters; the range and marginal standard deviation hyperparameters control spatial variation, and precision and correlation hyperparameters of the AR1 temporal component control how the spatiotemporal effect evolves through time. We applied penalised‐complexity priors for these hyperparameters as they are informative flexible priors that reduce overfitting (Simpson et al. 2017). To prevent overfitting, spatial range collapse and variance explosion in the spatiotemporal random effect, we constrained the model's hyperparameters by fixing the spatial range and the marginal standard deviation to constant values. The values were set a priori based on mobility of the fish species, empirical variograms and based on the observed posterior values for these hyperparameters from null models fit with only an intercept and a spatiotemporal random effect. The hyperparameters were fixed with a range of 60 km and variance (SD) of 0.35 for the sandeel model, and a range of 300 km and variance (SD) of 0.3 for the sprat model.

By linking the mesh nodes across each time step, INLA can estimate the spatial dependence across time. For this we used a first order auto‐regressive (AR1) process with a time step for every year fit as a separable spatiotemporal random effect. The spatiotemporal fields capture structured residual variation not explained by environmental covariates included in the model. One key unmodelled factor potentially contributing to spatio‐temporal variation is fishing pressure (F) from the stock assessment model. As a preliminary test, we examined whether the temporal trend in the spatiotemporal random effect for each sandeel management area correlated with ICES estimates of fishing mortality. This provides insight into how much of the residual structure in the spatial field might reflect anthropogenic impacts.

As we joined survey datasets together that have a mismatch in temporal coverage, we performed sensitivity analyses to check the effect of this on the model. We compared the start time of the sandeel dredge survey and HERAS in the sandeel and sprat models, respectively, on the trend of the spatiotemporal random effect (Figure S3). Sandeel area 3r was the only component to show a change in spatiotemporal random effect following the introduction of extra data into the model; given this limited extent across both models we consider the effect negligible and continued without accounting for this.

## 3 Results

### 3.1 Model Validation

The sandeel model produced robust predictions, which were also consistent with other modelling efforts. For the train‐test split validation dataset, the model predictions closely matched observed biomass values, with a normalised root mean square error (NRMSE) of 0.002. Spatial predictions were broadly consistent with Langton et al. (2021), with a correlation coefficient of 0.75 (*p* < 0.001) between model outputs (Figure 2). Compared to the Langton model, the INLA model estimated a higher biomass index in most areas, except for the western Dogger Bank, adjacent southern sandbanks and parts of the Moray Firth. Notably, the INLA model predicted higher biomass than the Langton model in most of the core fishing grounds (Figure 2C), which are often used as a proxy for areas with high sandeel abundance.

**FIGURE 2:**
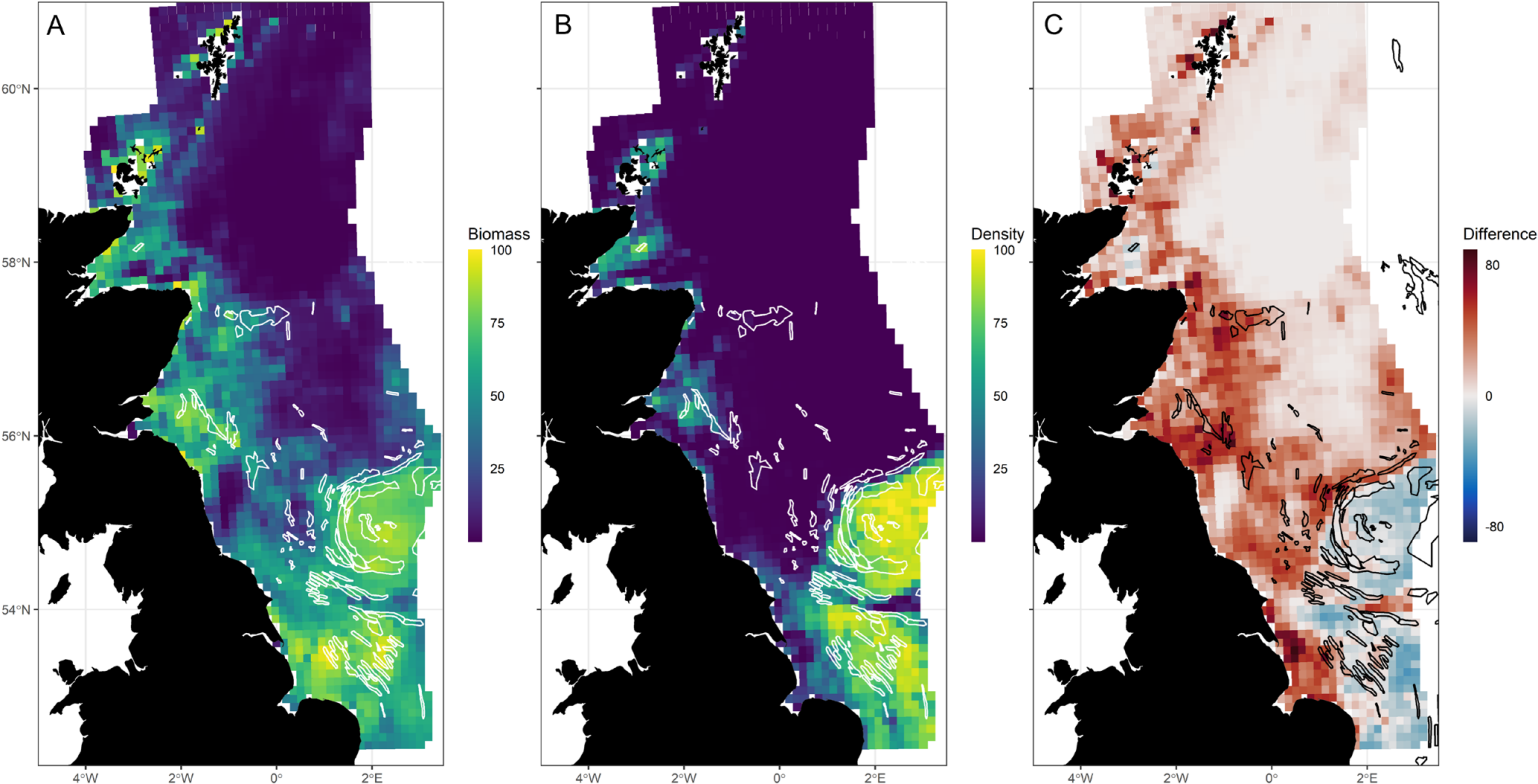
Comparison of predictions of (A) sandeel biomass from the INLA model with (B) density from the Langton model. (C) shows the difference between them as the Langton predictions subtracted from the INLA predictions after both datasets have been converted to a log scale and each standardised between 0 and 100 to make them comparable. Areas in red were predicted to have higher biomass in the INLA model and areas in blue higher abundance in the Langton model. Core fishing grounds from Jensen et al. (2011), which are a proxy for sandeel habitat, are overlaid in white for (A) and (B) and black for (C).

Temporal comparisons with ICES stock assessments showed the highest alignment in areas 3r (Skagerrak, northern and central North Sea) and 2r (central and southern North Sea), with correlation coefficients of 0.75 (*p* < 0.001) and 0.36 (*p* = 0.11), respectively (Figure 3A). Correlations were weaker in area 4 at 0.20 (*p* = 0.38), and negative (though not significantly so) in area 1 at −0.21 (*p* = 0.36). Fixed effect estimates revealed the highest biomass in dredge surveys (posterior mean = −3.25), followed by Q3 IBTS (posterior mean = −4.21), Q1 IBTS (posterior mean = −5.70), and BTS, which showed substantially lower biomass (posterior mean = −10.24) (Figure 4).

**FIGURE 3:**
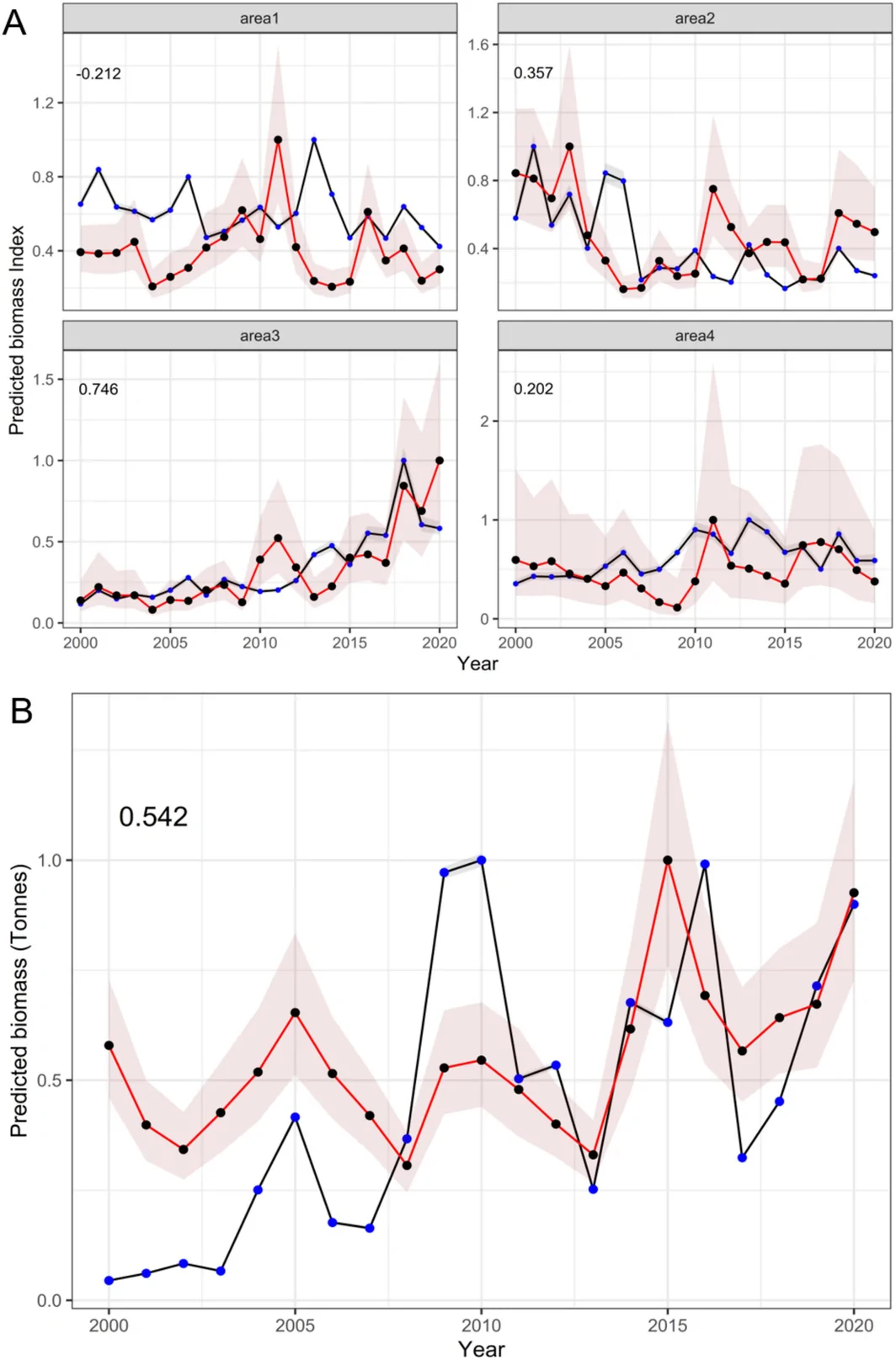
Comparison between time series of biomass predicted our models (black) with biomass estimates from the ICES stock assessments (red) for (A) sandeel areas 1–4 and (B) sprat. values are scaled between 0 and 2 to allow direct comparisons between the model outputs. Pearson's correlation coefficients are shown in the top left corner on each panel. The grey shaded bands represent the 95% Credible Intervals obtained from posterior sampling of the joint distribution from the INLA models, while the red shaded bands represent uncertainty in the stock estimates from the ICES stock assessment models.

**FIGURE 4:**
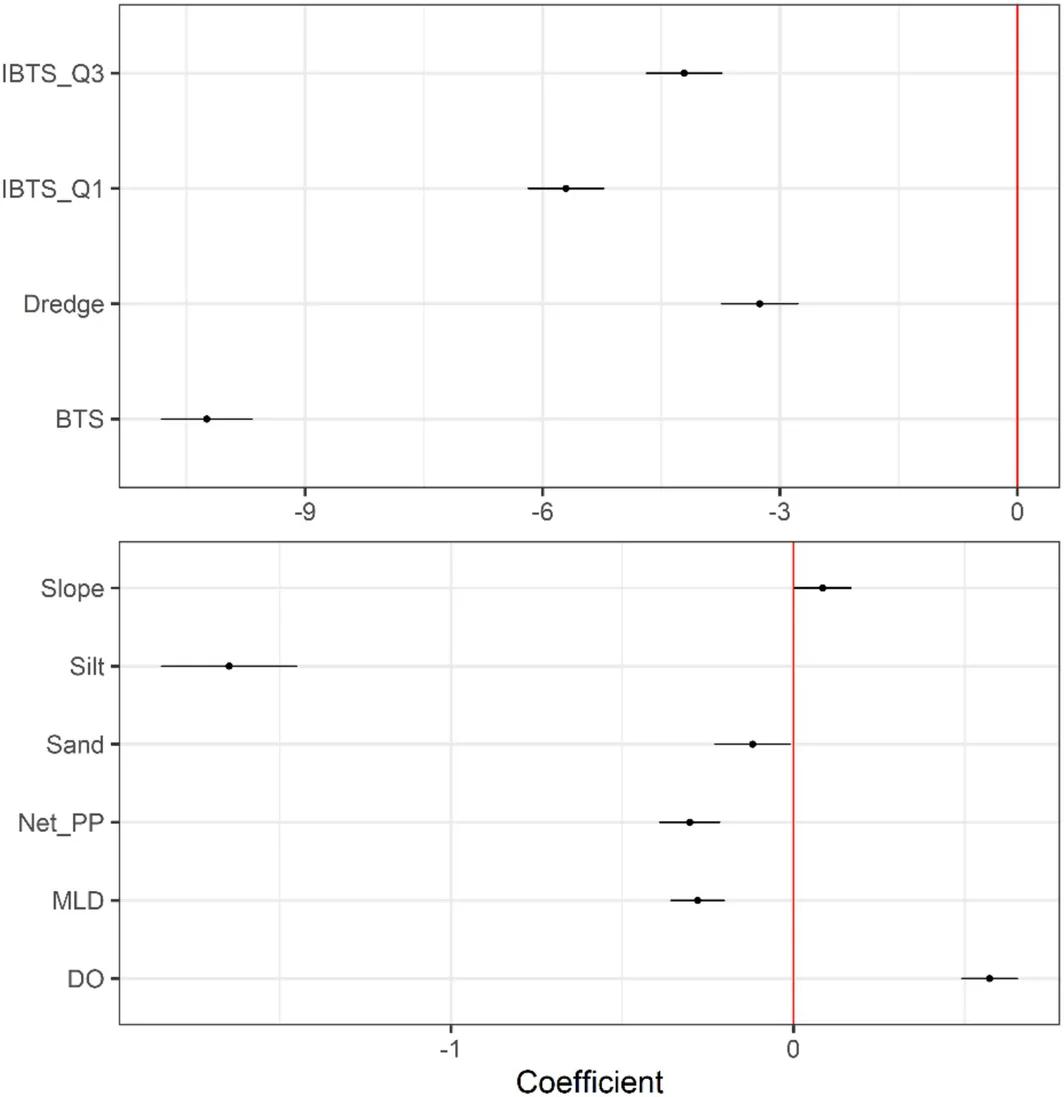
Fixed effect coefficient estimates from the sandeel model. Points represent the posterior mean while the horizontal bars represent the 95% credible interval. The vertical red line denotes 0. IBTS = International Bottom Trawl Survey, BTS = Beam Trawl Survey, Net_PP = Net Primary Productivity, MLD = Mixed Layer Depth, DO = Dissolved Oxygen.

The sprat model predictions closely matched observed values in the validation dataset, with a normalised root mean square error (NRMSE) of 0.003. Temporal estimates of sprat biomass were moderately correlated with ICES stock assessment estimates for the Skagerrak, Kattegat and North Sea, with a correlation coefficient of 0.555 (*p* = 0.009) (Figure 3B). Temporal patterns were broadly similar, though the model predicted lower biomass from 2000 to 2007 and substantially higher biomass in 2009–2010, with closer alignment between 2011 and 2020. Fixed effect estimates indicated the highest predicted sprat biomass in June (posterior mean = −0.89), followed by July (posterior mean = −1.49), August (posterior mean = −1.91) and September (posterior mean = −2.27). Biomass estimates were lower in the HERAS survey (posterior mean = −3.63) than in the IBTS (posterior mean = −2.93) (Figure 6).

### 3.2 Environmental Drivers

The final models are shown in full in Table 2. Sandeel biomass was negatively associated with silt content, sand content, net primary productivity and mixed layer depth, and positively associated with dissolved oxygen concentration and seafloor slope (Figure 4). Higher sandeel biomass was predicted at depths shallower than 75 m (Figure 6A). Sprat biomass showed a negative association with bottom water velocity and positive associations with seafloor slope and mixed layer depth (Figure 5). Predicted sprat biomass was highest at depths less than 200 m (Figure 6B) and at phytoplankton concentrations above 8 mmol m^−3^ (Figure 6C).

**TABLE 2:** The best retained model for each species.

Species	Model formula
sandeel	NPP + sand_per + silt_per + slope + MLD + DO + survey + f(depth) + f(space–time)
sprat	VEL + slope + MLD + survey + month + f(depth) + f(PHYC) + f(year) + f(space–time)

*Note:* Parentheses *f*(…) denote smooth terms modelled as random effects.

Abbreviations: depth, bathymetric depth; DO, seabed dissolved oxygen; MLD, mixed‐layer depth; NPP, surface net primary productivity; PHYC, surface phytoplankton concentration; sand_per, seabed sand percent; silt_per, seabed silt percent; slope, seabed slope; survey, survey ID.

**FIGURE 5:**
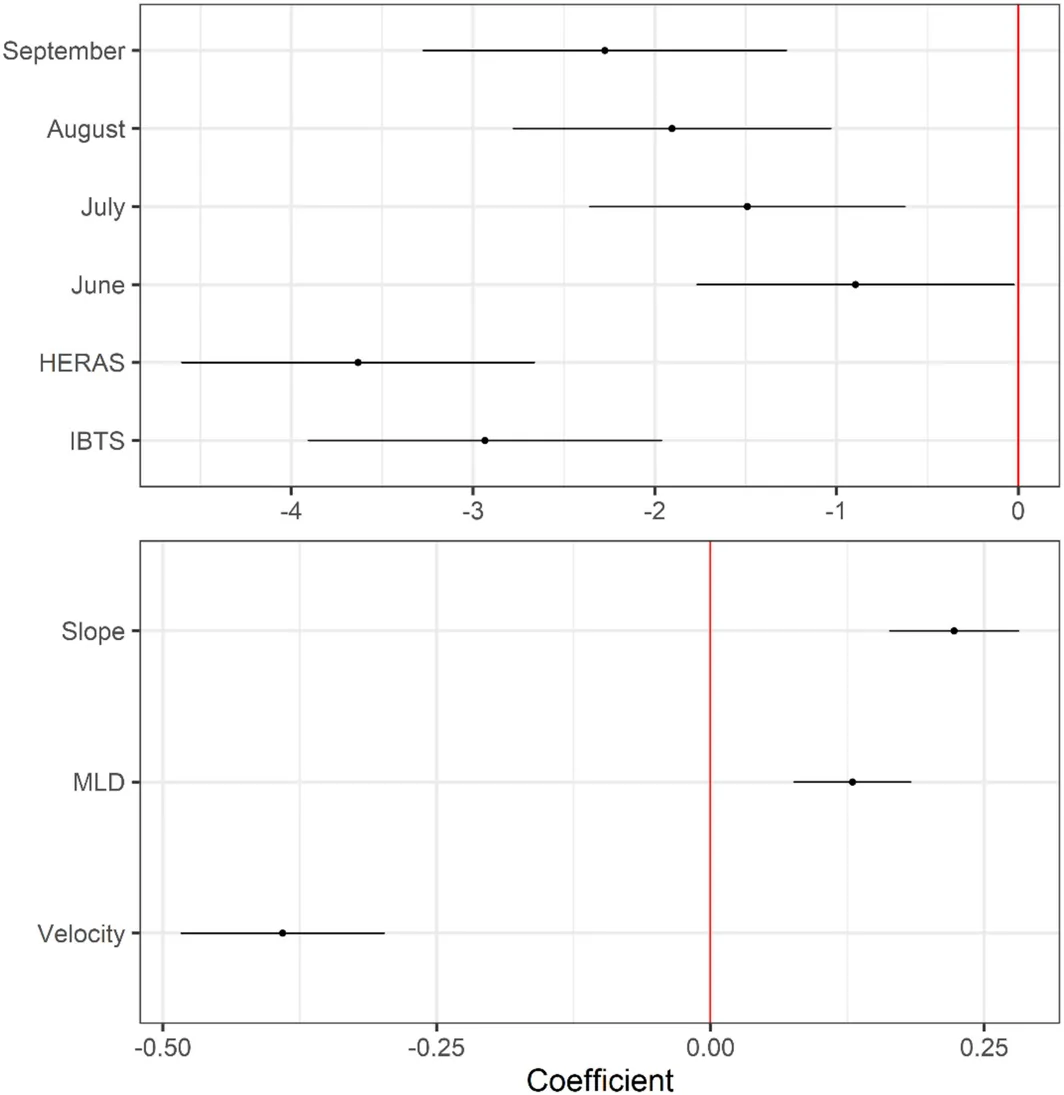
Fixed effect coefficient estimates from the sprat model. Points represent the posterior mean while the horizontal bars represent the 95% credible interval. The vertical red line denotes 0. HERAS = Herring Acoustic Survey, IBTS = International Bottom Trawl Survey, MLD = Mixed Layer Depth.

**FIGURE 6:**
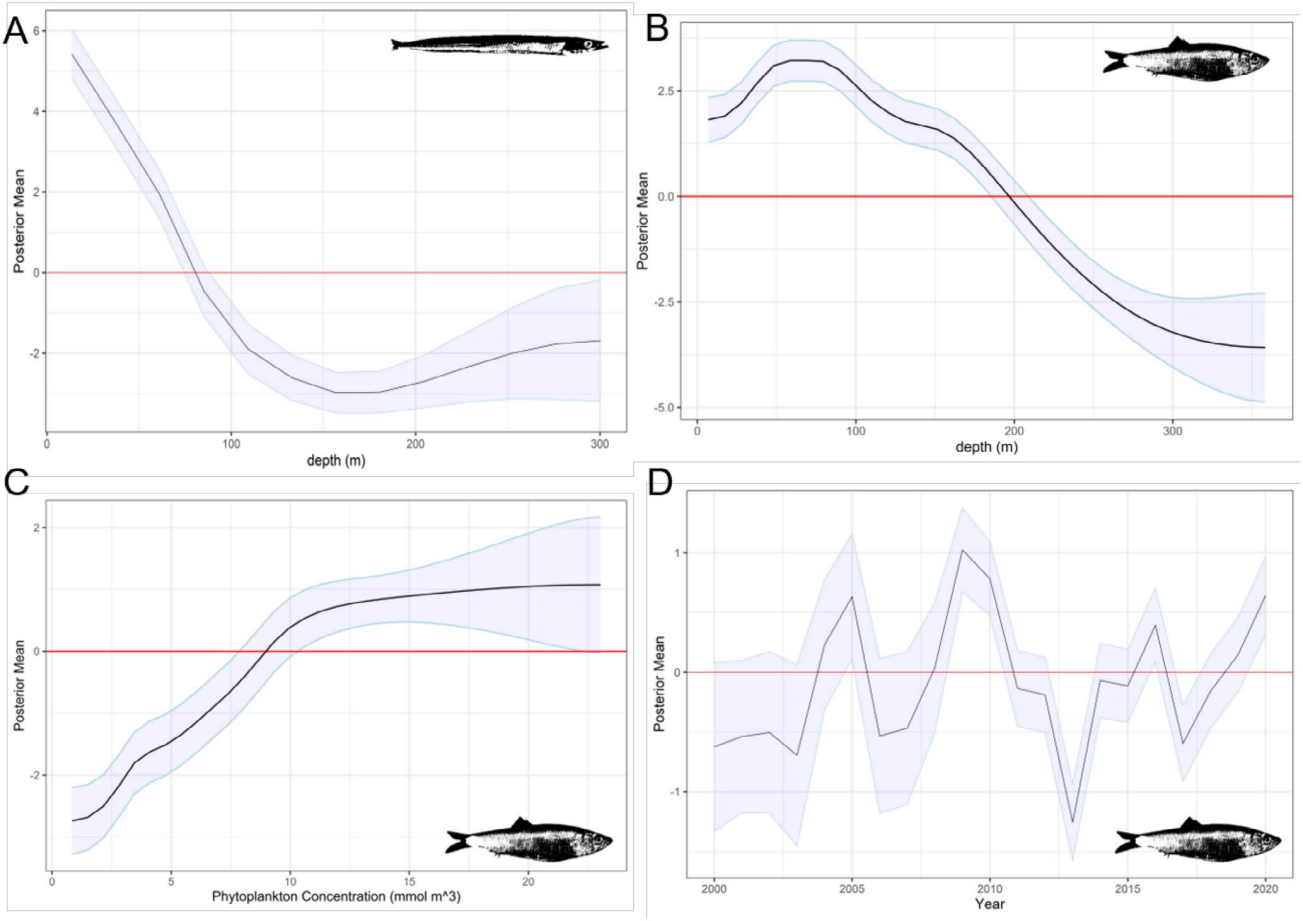
Smoothed non‐linear random effects showing the modelled relationship between (A) sandeel biomass and depth (B) sprat biomass and depth and (C) sprat biomass and phytoplankton concentration. (D) The AR1 effect for year for sprat. The red lines denote 0, where points above showing a positive effect and points below a negative effect.

### 3.3 Fine‐Scale Spatial–Temporal Predictions

Our model predicts that sandeel biomass is distributed throughout the main sandbank habitats of the North Sea region (Figure 7), broadly in alignment with core sandeel fishing grounds (Jensen et al. 2011). Our model predicted high biomass in all of the large fishing grounds, with only a few very small and isolated fishing grounds that were not predicted to contain sandeel biomass, and a decreasing gradient of sandeel biomass outside of the core fishing grounds (Figure 7).

**FIGURE 7:**
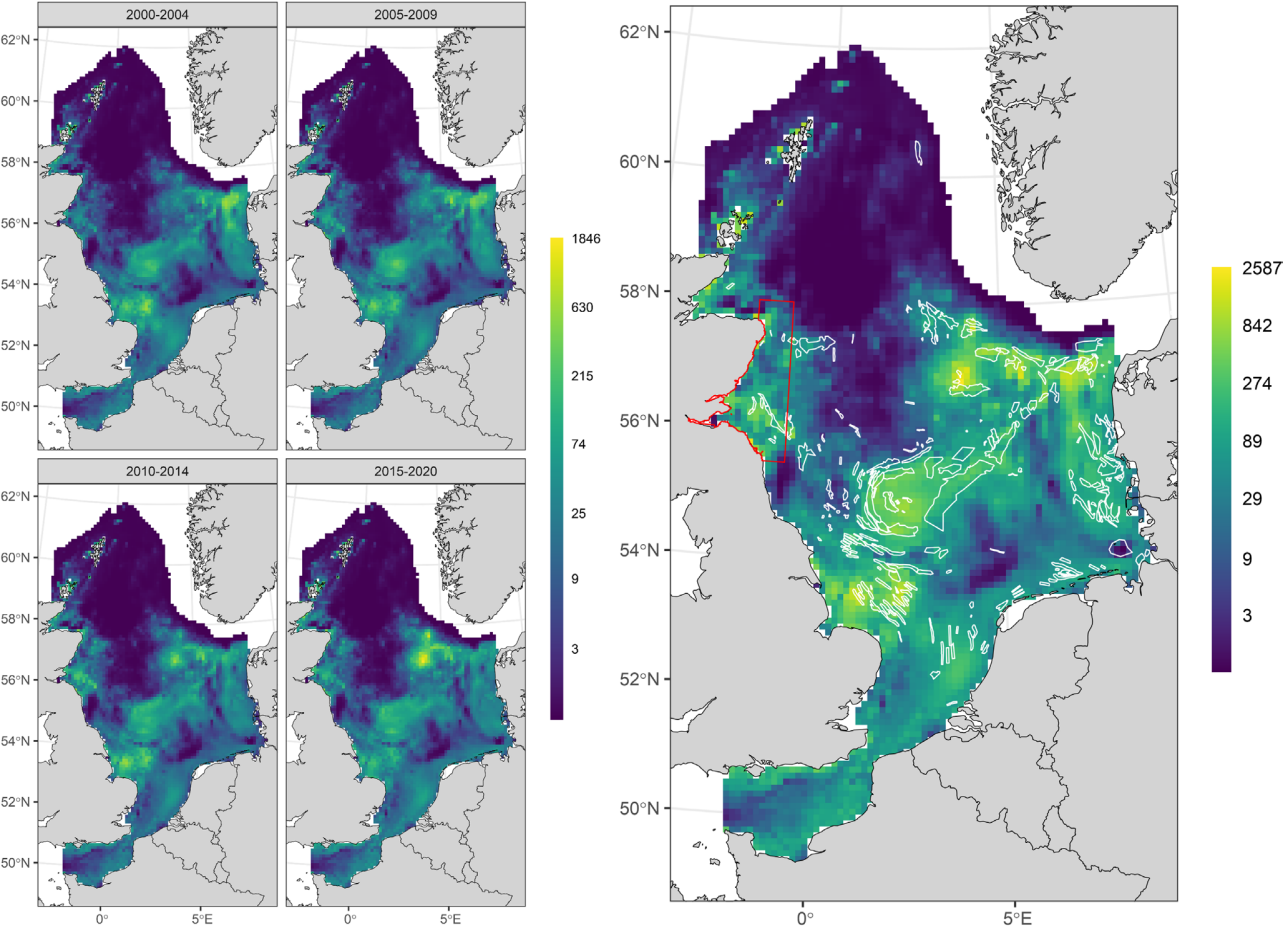
Predicted sandeel relative biomass (Left) summed into four five‐year periods over the full timeseries and (Right) Summed for the 21‐year time period White lines denote major sandeel fishing grounds from Jensen et al. (2011). The red line shows the area closed to sandeel fishing off of the East coast of Scotland. Biomass values are shown on log scale in kg per 10 km grid cell.

Temporal trends in sandeel biomass in each sandeel management area are shown in Figure 8A. All sandeel areas displayed interannual variation consistent with small pelagic fish boom and bust population dynamics. We also made predictions for area 5 (northern North Sea, Viking and Bergen banks) and area 7 (northern North Sea, Shetland), as well as the channel, which do not currently have stock assessments performed and thus these predictions represent the only estimate of regional sandeel population size in these areas. Area 5 was predicted to have very low but variable biomass over the time period, while area 7 initially decreased but then recovered at the end of the timeseries.

**FIGURE 8:**
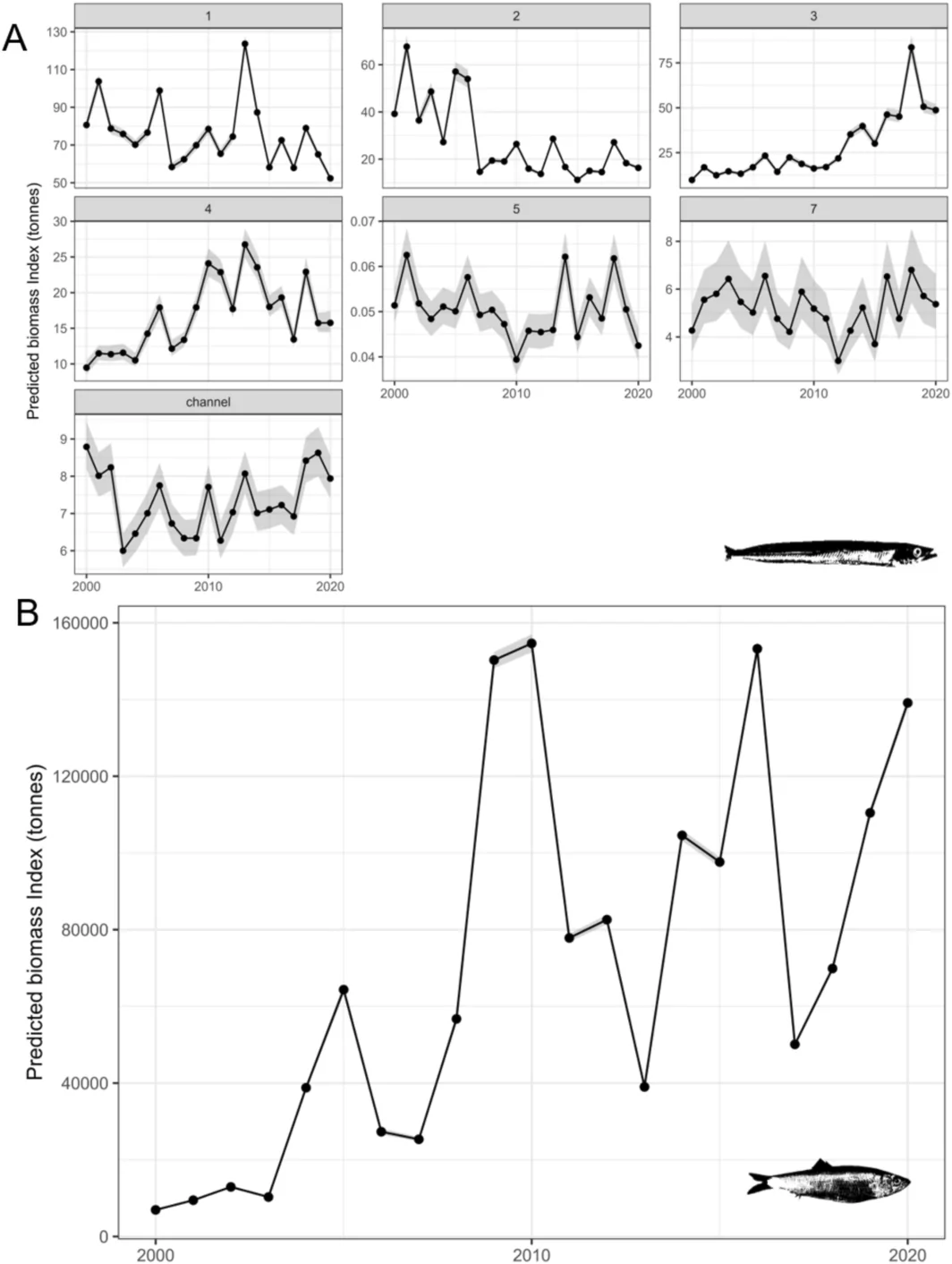
Timeseries of biomass for (A) sandeel across the 7 Management Areas with additional category for the English Channel and (B) sprat across the greater North Sea and the Scottish coast north of the firth of forth. Note the sandeel model is relative biomass. The grey bands represent the 95% Credible Intervals obtained from posterior sampling of the joint distribution.

There are clear trends in the spatial distribution of sprat biomass over the time period covered by our study (Figure 9). The model predicts that sprat biomass is initially mostly distributed in the south‐eastern North Sea, extending further west over time towards the UK coast, with population increases also seen off the East coast of Scotland, concentrated in southern Moray Firth and south around the headland in Aberdeen Bay (Figure 9). The central southern region sees an expansion northward, with a noticeable absence from the Dogger Bank area. Our results predict that sprat were found as far north as Scotland at the beginning of the assessment period, but they have increased in density over the past two decades. Looking at the overall temporal trend, sprat biomass tended to increase across the North Sea over the 21‐year study period with an oscillating cycle of higher and lower biomass roughly every 3–4 years (Figure 6D; Figure 8B).

**FIGURE 9:**
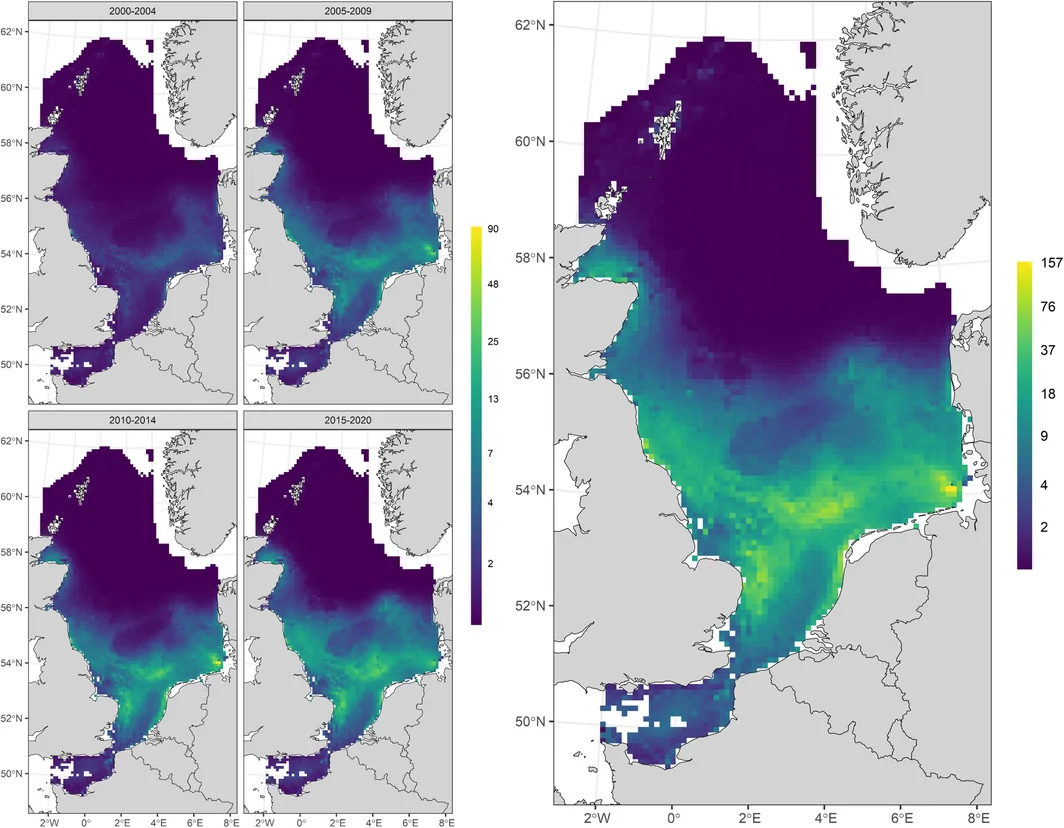
Predicted sprat biomass (Left) summed into four five‐year periods over the full timeseries and (Right) summed for the 21‐year time period. Biomass values in tonnes per 10 km grid cell are shown on log scale.

### 3.4 Spatial Temporal Effect

Residual spatial variation was evident in both the sandeel and sprat models and was effectively captured by the spatiotemporal random effects. The spatiotemporal effect in the sandeel model varied over time (Figure 10A,B), predicting higher than average biomass over the southern and western North Sea (sandeel areas 1r and 2r, respectively) at the beginning of the timeseries. This reversed over time and in later years the spatiotemporal effect was highest over northern central North Sea (sandeel areas 3r and 4), with the effect changing to negative over sandeel areas 1r and 2r. For sprat, the spatiotemporal effect had a relatively stable distribution across the study period; however, there was a considerable strengthening of the magnitude of the effect throughout the timeseries (Figure 10C,D). The sprat spatiotemporal effect had a distinct north–south distribution, with positive values in the southern North Sea and negative values in the north. Annual maps of smoothed random fields for both species are provided in the Supporting Information (Figure S3, S4).

**FIGURE 10:**
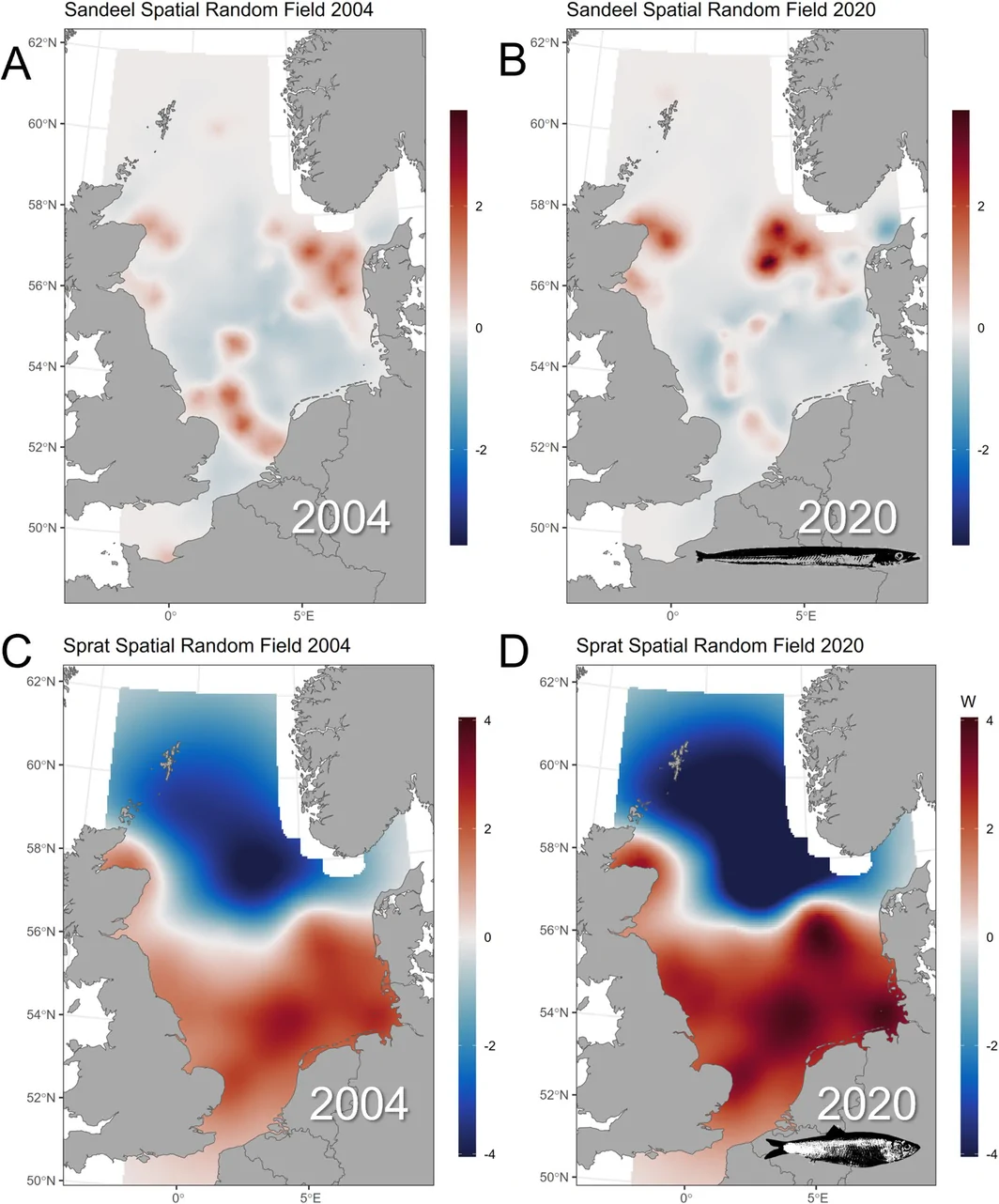
Smoothed random field (dimensionless on the log scale) of the spatiotemporal effect for sandeel for (A) 2004 and (B) 2020, and for sprat for (C) 2004 and (D) 2020. The random field approximates the residual variation in the model, with red areas containing above average and blue areas below average residual biomass. Non‐random spatially structured colours suggest spatial autocorrelation in the data not fully accounted for by the environmental covariates. Notice the colour bars are on different scales for each species.

Sandeel Area 4 was the only region to show a moderately high negative correlation between the spatiotemporal effect in the sandeel model and fishing pressure (*r* = −0.575, *p* < 0.001) (Figure 11). The spatiotemporal effect increases from 2000. There is a distinct change in pattern with a decrease in the effect from 2012. The spatiotemporal effect decreases further as fishing pressure increases until 2018, after which fishing pressure drops and the spatiotemporal effect increases slightly. In sandeel areas 1 (*r* = 0.84, *p* = 0.01) and 2 (*r* = 0.48, *p* = 0.05) the spatiotemporal effect temporal trend was moderately positively correlated with F for the respective management areas. There was no significant correlation for area 3 (*r* = 0.04, *p* = 0.67). For sprat there was no correlation between the spatial effect and fishing pressure.

**FIGURE 11:**
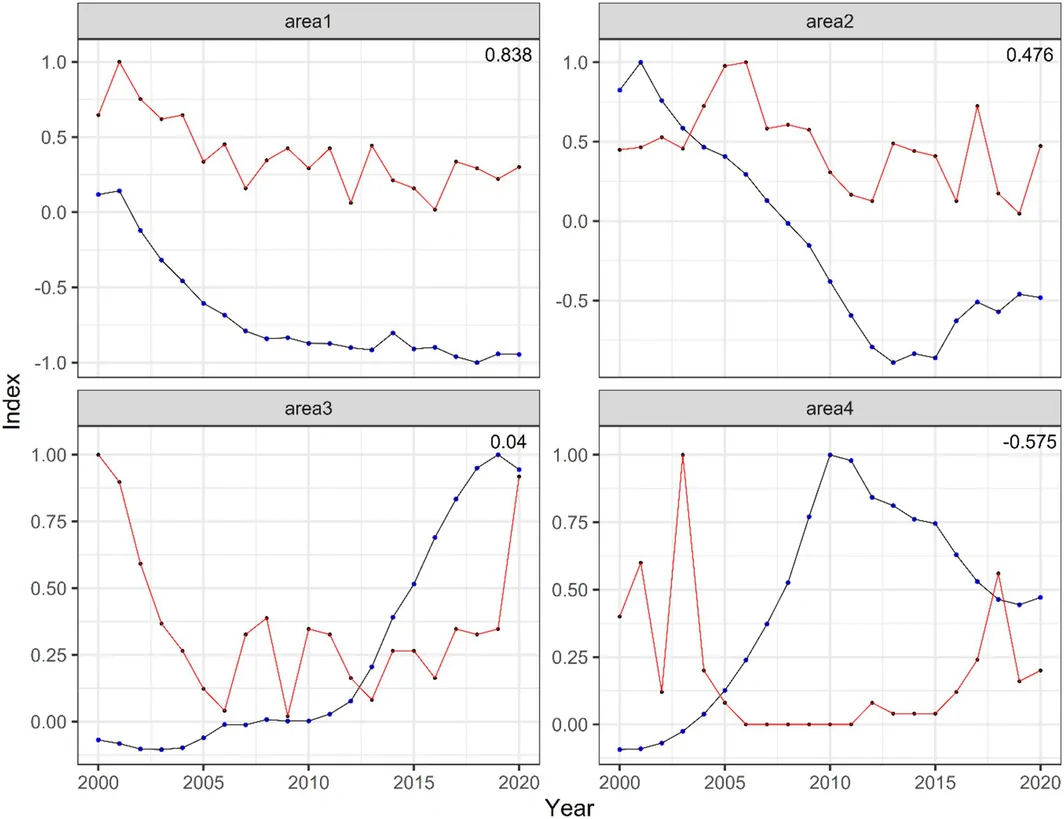
Comparison of the spatiotemporal effect temporal trend (black) with fishing pressure from ICES stock assessment (red) for four sandeel areas that have a stock assessment conducted. Pearson's correlation coefficient shown in the top right corner.

## 4 Discussion

Detailed forage fish distribution data across large spatial and temporal scales in the North Sea are vital to future work understanding predator dynamics, the ecosystem effects of commercial fishing, marine spatial planning and impacts from climate change. These fish are not fully sampled across their full range by any specific survey and thus require appropriate modelling that can join surveys together and properly account for sources of uncertainty. Here, we gathered available large scale survey data and employed modern Bayesian modelling techniques using R‐INLA to join these surveys together and produce detailed fish biomass estimates across space and time over the past two decades. We were able to produce robust models that were moderately correlated with other analyses; however, this varied by region and time (ICES 2022a; Langton et al. 2021). We further discuss instances where our predictions diverged from other models and methods to improve the methodological framework. In addition, by accounting for catchability and pooling with the higher accuracy dredge data, bottom trawl data was able to be included in a model that produced sensible predictions for sandeel. This model, in addition to the sprat model, provide biomass estimates at more widespread spatial and temporal scales than previous work (Jensen et al. 2011; Langton et al. 2021; Lindegren et al. 2022), filling a vital knowledge gap of the North Sea marine ecosystem and lay the foundation for future ecological work.

Importantly our model makes predictions for areas where no sandeel stock assessments or commercial catch occur, providing novel estimates of the spatial distribution and temporal trends of sandeel around the Shetland and Orkney Islands which are important to seabirds and cetaceans (Waggitt et al. 2020), as well as sandeel area 5, off the West coast of Norway, and the eastern English Channel. Results from our model showed that hotspots of sandeel biomass occurred in areas already identified as major fishing grounds, a proxy for sandeel habitat (Jensen et al. 2011), however, there were also areas adjacent to these that were predicted to contain moderate levels of sandeel biomass that are likely important to local predators. The low predicted biomass for sandeel area 5 across the past two decades agrees with monitoring work by Norwegian Institute of Marine Research (IMR) (Falkenhaug et al. 2016). The sandeel fishery in the Shetland region closed in the early 2000s because the low variable catch became uneconomical (ICES 2022a). This region was also the first to see seabird breeding season food shortages in the North Sea as far back as the 1980s (Monaghan et al. 1989). Having information on the distribution and trend of vital prey in this area is a large benefit and could hopefully improve understanding and management of both prey and predators in the area.

The dredge survey caught the highest amount of sandeel, as expected, followed by the summer IBTS. The winter IBTS catches were lower, this is likely due to the sandeel being buried in the sediment (Winslade 1974). Finally, the BTS catches were markedly lower than the other surveys, likely due to its smaller nets with a lower vertical opening. The sprat model predicted lower sprat biomass in the HERAS survey than in the Q3 IBTS. This may seem counterintuitive as HERAS has higher values of biomass when sprat are present. However, it also has a substantially higher proportion of true zeros (absences) owing to the very high density of acoustic data points. The results for the sprat datasets were less intuitive and this is likely caused by the complexity of joining two fundamentally different survey types (acoustic vs. bottom trawl). The results for the sandeel data are relatively straight forward, showing that the dedicated survey caught the most sandeel. However, they also show that the summer IBTS survey caught the next most amount of sandeel and when modelled properly can provide important information on the species.

Comparing our sandeel predictions to the ICES stock assessment was not straightforward, as the stock assessment includes catch data and considers all sandeel lengths, whereas our model included sandeel data up to and including 16 cm, which was chosen to represent the maximum size of prey edible by chicks of the medium sized auks and black‐legged kittiwake which dominate the seabird community in this region (Thaxter et al. 2013). However, there was strong agreement for certain areas. Area 3 had a very strong correlation while area 2 had a moderate correlation, showing a similar general pattern. Area 4 and 1 had low overall temporal correlation to the stock assessment timeseries, however, there were certain periods with strong similarities, such as the first half of the timeseries for area 4 or 2015 to 2020 in area 1. It is not clear why the strength of this temporal relationship varies between areas.

Sandeel Area 3 was the only sandeel management area to show a clear upward trend in population size (Figure 8). In 2011, Norway implemented an area‐based management plan that subdivides Area 3 to provide finer scale management and in‐season dynamic advice (Johannessen and Johnsen 2015). We were not able to test the sub‐management area population dynamics but given the limited post‐settlement connectivity of sandeel this may suggest finer scale management is beneficial for the species. However, Area 3 is relatively small compared to the rest of the North Sea and scaling this up for the whole region may not be possible due to the cost involved. This also raises questions related to the impacts of fishing effort displacement in the case of closures, as total allowable catch is set for the whole of each management area leading to a potential displacement of commercial fishing activity from closed to open parts of the area (ICES 2022a). A large part of Area 4 off the coast of Scotland has been closed since 2000, with the commercial sandeel fishing industry around the Shetland Islands (Sandeel Area 7) becoming economically unviable in the early 2000s and a portion of the Dogger Bank (Sandeel area 1) closed since 2022. Now since 2024, these have been extended and codified into law as the entire UK EEZ has been closed to sandeel fishing, thus while there was a high risk of displacement of fishing pressure in Area 4 over the past 2 decades, the recent UK closure has removed that risk, but now creates a strong risk for fishing effort displacement in Sandeel area 1, which has a large section inside the UK EEZ (Figure 1).

Our estimate of sprat biomass was moderately correlated to the stock assessment model outputs, which is to be expected as both HERAS and IBTS Q3 survey data are components in both models. However, our model does not contain catch data and does not cover the Kattegat or Skagerrak areas which since 2019 have been included in the greater North Sea sprat assessment, and data integration differs between the modelling frameworks. These differences may be a source of divergence between our model trends and that of the stock assessment. The IBTS and the HERAS surveys both collect data on sprat; however, they have very different methodologies and survey differing portions of the water column. This is evident as the raw biomass values in the data display different temporal trends across the timeseries. This large variation between surveys has likely led to uncertainty of the true population size. More advanced data integration techniques would be highly advantageous for modelling sprat distributions and reconciling these informative but fundamentally different datasets. While the sandeel model exhibited less variation between survey types, its predictive power would likely also be enhanced by adopting advanced integration methodologies. In this study, we assumed that biomass‐environment relationships are invariant across gear types, even if gear‐specific catchability differs. Including gear type as a fixed effect provided a pragmatic and statistically sound way to control for baseline differences in absolute efficiency, thereby permitting data pooling. Future research using this spatiotemporal modelling framework should focus on implementing formal data integration methods to explicitly resolve these gear discrepancies (Paradinas et al. 2023).

Our model estimates that sprat biomass has been increasing across their known hotspots with a steady expansion north over the past 20 years. This could be either due to slow recovery from past overfishing in the 1980s, with competition forcing fish to occupy sub‐optimal habitat on the edge of their northern range or expansion of favourable conditions. We observed the spatiotemporal effect north–south boundary pushes slowly north and east. This could potentially be a range expansion northward with climate change. We found no correlation between the spatiotemporal effect (residual variation in biomass) and fishing pressure estimated from the stock assessment. This suggests fishing may not be the main driver of sprat stock dynamics in the North Sea over the past two decades. However, this evidence does not enable us to comment on the effects of sprat fishing on intra‐annual food availability to dependent predators such as seabirds. Thus, our selection of environmental variables was also not able to capture the driver of this range expansion and further work is needed to investigate this.

Comparing the temporal trend of the spatiotemporal effect (residual spatiotemporally structured variation) of the sandeel model with fishing pressure found a negative correlation only in Area 4. Here the spatiotemporal effect increases from 2000 to 2010; however, the direction of change (increase) does not signify an increase in pressure, only a change in the effect size of the spatiotemporal effect (log scale). This pattern aligns with the designation of the sandeel fishing closed area off the coast of Scotland in 2000 and the complete cessation of fishing in the remainder of Area 4 in 2005. There is a distinct change in pattern with a decrease in the spatial effect from 2012, which coincides with the resumption of fishing in Area 4 outside of the closed area. Sandeel area 4 has been identified as the most isolated subpopulation through otolith analysis, biophysical models and genetic analysis (Jiménez‐Mena et al. 2020; Wright et al. 2018). Area 4 has also been identified as having the slowest growth rate and lowest productivity of the North Sea regional populations (Boulcott and Wright 2011; Rindorf et al. 2016). While this comparison is only a simple broad‐scale correlation, these factors could potentially explain why this was the only region we observed the spatiotemporal random effect to be correlated with fishing pressure—as the reproductive isolation and low productivity may increase the vulnerability to localised overfishing—and certainly warrants further study.

Our estimation of sandeel relationships with seabed characteristics generally agrees with previous studies (Holland et al. 2005). Counterintuitively, we found a small negative sandeel biomass association with percentage sand content. It is likely that the sediment groupings used in our model were too broad, with Holland et al. (2005) noting sandeel avoidance of fine‐grained sand and preference for coarser‐grained sand. Modern methods using underwater video and multi‐beam echosounders have been recently developed on local scales to accurately understand sandeel sediment preferences (Baker et al. 2024; O'Kane et al. 2026), however, such methods have not been employed for the larger North Sea region. Future novel methods to further elucidate the sediment associations of bentho‐pelagic fish such as sandeel will likely be of great benefit to the management of the species. We identified bottom dissolved oxygen as an important driver of sandeel biomass. Studies of closely related *Ammodytes tobianus* have identified that seasonal bottom oxygen deficiencies have been increasing but are annually variable, possibly limiting available sandeel habitat (Behrens et al. 2009). This may negatively impact sandeel in the future as shelf seasonal oxygen depletion is projected to increase with increased stratification due to climate change (Holt et al. 2010).

Sprat are known to be able to tolerate a wide range of environmental conditions, and it has been difficult to link sprat to particular habitat (Engelhard et al. 2013). The strong spatiotemporal random effect indicates the distribution is not fully described by the selected variables and other effects may be influencing the distribution of sprat across the North Sea. While we cannot at present describe these unknown drivers of the sprat population, the random field successfully captured the effect across space and time and significantly improved our predictions.

Future modelling work will most certainly improve as finer‐scale environmental raster layers become available, especially important for animals such as sandeel that are highly habitat specific. The data pooling methods we employed would likely benefit greatly from applying novel combined species distribution modelling techniques (Isaac et al. 2020), that can join datasets together in a more sophisticated way than we were able to do here. These methods can account for the observation process as well as the biological process, and will likely be a huge leap forward for studying these important but understudied fish (Chadwick et al. 2023; Paradinas et al. 2023).

## 5 Conclusion

Data on forage fish distributions is a key missing link in studying the ecosystems of the North Sea. Here we have gone some way towards filling this gap by creating fine‐scale predictions of sandeel and sprat biomass at the North Sea scale across the past two decades. We show that, contrary to common assumptions for sandeel, bottom trawl survey data can inform models that produced sensible results, with the advantage that resulting predictions are not made for unobserved regions. The modelled datasets are openly available to inform future work on the North Sea ecosystem, such as the trophic dynamics of seabirds and other predators. Our work lays down a foundational step forward for understanding the North Sea pelagic ecosystem, across ecologically relevant spatial and temporal scales.

## Author Contributions

**Sylvan Benaksas:** conceptualization (equal), data curation (lead), formal analysis (lead), methodology (equal), software (equal), validation (lead), writing – original draft (lead). **Christopher Lynam:** conceptualization (equal), funding acquisition (equal), methodology (equal), supervision (equal), writing – review and editing (equal). **Francis Daunt:** conceptualization (equal), methodology (equal), supervision (equal), writing – review and editing (equal). **Chibuzor Christopher Nnanatu:** formal analysis (supporting), methodology (equal), software (equal). **Jeroen van der Kooij:** data curation (supporting), formal analysis (supporting), writing – review and editing (supporting). **Thomas J. Webb:** conceptualization (equal), formal analysis (equal), funding acquisition (equal), methodology (equal), supervision (equal), writing – review and editing (equal).

## Funding

This research was funded by the UK Natural Environment Research Council through the Adapting to the Challenges of a Changing Environment (ACCE) doctoral training program grant number NE/S00713X/1, in partnership with CEFAS through the Seedcorn programme.

## Conflicts of Interest

The authors declare no conflicts of interest.

## Supporting information


**Figure S1:** Map of the mesh used for the sandeel model for approximating the random field covering the North Sea region, enclosed by the blue line. The mesh boundary is coarsely clipped to the North Sea coastline to avoid estimation across land masses. There is an outer boundary enclosed by the black line that contains large triangles to avoid edge effects.
**Figure S2:** Map of the mesh used for the sprat model for approximating the random field covering the North Sea region, enclosed by the blue line. The mesh boundary is coarsely clipped to the North Sea coastline to avoid estimation across land masses. There is an outer boundary enclosed by the black line that contains large triangles to avoid edge effects.
**Figure S3:** Annual spatiotemporal random fields for the sandeel model. W is the spatial effect parameter and is on the log scale.
**Figure S4:** Annual spatiotemporal random fields for the sprat model. W is the spatial effect parameter and is on the log scale.

## Data Availability

All data used in this paper are publicly available, with sources provided in the main text. Modelled predictions of fishbiomass—comprising one dataset for sprat and two for sandeel (the length‐restricted model and the full‐length distribution model)—as well as the accompanying code are available at https://orda.shef.ac.uk/articles/dataset/North_Sea_Forage_Fish_Distributions/28451972.
