# Supplementary material for: Seabird Prey in Space and Time: Combining Multiple Datasets to Model the Distribution of Sandeel (Ammodytes marinus) and Sprat (Sprattus sprattus) in the North Sea
Source: Ecol Evol. 2026 Oct 29;16(11):e74570. doi: 10.1002/ece3.74570 (PMC13717606; doi:10.1002/ece3.74570)
Supplement: Supplementary file 1 [file ECE3-16-e74570-s001.docx]

Supplementary Material for Seabird prey in space and time: Joining multiple datasets to model the distribution of sandeel (*Ammodytes marinus*) and sprat (*Sprattus sprattus*) in the North Sea

Sylvan Benaksas^1*^, Christopher Lynam^2^, Francis Daunt^3^, Chibuzor Christopher Nnanatu^2#^, Jeroen van der Kooij^2^, Thomas J. Webb^1^

^1^Ecology & Evolutionary Biology, School of Biosciences, University of Sheffield, Sheffield, United Kingdom

^2^Centre for Environment, Fisheries and Aquaculture Science, Lowestoft Laboratory, Lowestoft, Suffolk NR33 0HT, United Kingdom

^3^UK Centre for Ecology & Hydrology, Bush Estate, Penicuik, Midlothian, EH26 0QB, UK

^#^Current affiliation: WorldPop, School of Geography and Environmental Science, University of Southampton, Southampton, SO17 1BJ, UK

*Corresponding author Inland Fisheries Ireland, Dublin, Ireland; Email: [sylvan.benaksas@fisheriesireland.ie](mailto:sylvan.benaksas@fisheriesireland.ie)

Prior Specification

The spatial random field was modeled using a continuous Matérn covariance structure via the Stochastic Partial Differential Equation (SPDE) approach. Penalized Complexity (PC) priors were utilized to establish the hyperparameters. To prevent overfitting and maintain model stability, the spatial practical range and marginal spatial standard deviation were fixed constants for both models: 60 km and 0.35 for the Sandel model, respectively, and 300 km and 0.30 for the Sprat model, respectively. To capture temporal dynamics, the spatial random field was extended into a spatiotemporal process for both species by replicating it across time steps using a first-order autoregressive AR(1) structure. Penalized Complexity (PC) priors were assigned to the temporal hyperparameters. Specifically, a PC precision prior was used for the precision, setting the probability that it exceeds 1 to 5%. For the temporal autocorrelation parameter, *rho*, a PC correlation prior was implemented, specifying a base model of independence with a 50% probability that the correlation exceeds 0.5.

All fixed effects we fitted with gaussian priors with a mean of 0 and a precision of 1. Penalized Complexity (PC) priors were applied to regulate the smoothness and variability of 1D SPDE effects. To maintain structural stability and prevent over-fitting of the of the 1D SPDE effects, the practical range of the depth in the sandeel model was fixed to a constant 15 units on the mean-centred scale fitted with 15 knots, and 12 units for the sprat depth and phytoplankton concentration effects, both fitted with 35 knots. These values cover most of the covariate space and prevent very wiggly relationships. For the marginal standard deviation (sigma), a PC prior was specified such that the probability of the standard deviation exceeding 0.10 is 0.01% in the sandeel depth, sprat depth and sprat phytoplankton effects. Additionally, a sum-to-zero constraint (constr = TRUE) was enforced on the 1D fields to ensure identifiability against the group-specific intercepts. For the sprat global yearly temporal AR(1) effect, penalized Complexity (PC) priors were assigned to the temporal hyperparameters. Specifically, a PC precision prior was used for the precision, setting the probability that it exceeds 1 to 0.1%. For the temporal autocorrelation parameter, *rho*, a PC correlation prior was implemented, specifying a base model of independence with a 70% probability that the correlation exceeds 0.7, tighter priors were provided for this effect to avoid confounding with the space-time random effect.

Figure S1. Map of the mesh used for the sandeel model for approximating the spatial random field covering the North Sea region, enclosed by the blue line. The mesh boundary is coarsely clipped to the North Sea coastline to avoid estimation across land masses. There is an outer boundary enclosed by the black line that contains large triangles to avoid edge effects.

Figure S2. Map of the mesh used for the sprat model for approximating the spatial random field covering the North Sea region, enclosed by the blue line. The mesh boundary is coarsely clipped to the North Sea coastline to avoid estimation across land masses. There is an outer boundary enclosed by the black line that contains large triangles to avoid edge effects.

Figure S3. Annual spatial random fields for the sandeel model. W is the spatial effect parameter and is on the log scale

Figure S4. Annual spatial random fields for the sprat model. W is the spatial effect parameter and is on the log scale
